# Highly Breathable and Abrasion-Resistant Membranes with Micro-/Nano-Channels for Eco-Friendly Moisture-Wicking Medical Textiles

**DOI:** 10.3390/nano12173071

**Published:** 2022-09-04

**Authors:** Yue Zhang, Xing Li, Hong-Yang Wang, Bo-Xiang Wang, Jia Li, De-Hong Cheng, Yan-Hua Lu

**Affiliations:** 1 School of Chemical Engineering, Liaodong University, Dandong 118003, China; 2 Liaoning Provincial Key Laboratory of Functional Textile Materials, Liaodong University, Dandong 118000, China; 3 School of Textile Science & Engineering, Tiangong University, Tianjin 300387, China; 4 Tianjing Fire Science and Technology Research Institute of MEM, Tianjin 300381, China

**Keywords:** eco-friendly, micro-/nano-fibrous membranes, directional moisture transport, breathable and moisture-permeable, abrasion-resistant

## Abstract

One-way water transport is a predominant feature of comfortable textiles used in daily life. However, shortcomings related to the textiles include their poor breathability and durability. In this study, low-cost and eco-friendly PLA/low-melt (polylactic acid) LMPLA-thermoplastic polyurethane (TPU) membranes were fabricated through a needle punch/hot press and electrospinning method. The micro-/nano-channels, used for the first time, endowed the composite membranes with robust, breathable, moisture-permeable, and abrasion-resistant performance. By varying the nano- layer thickness, the resulting 16–40 μm membranes exhibited excellent one-way water transport, robust breathability and moisture permeability, and good abrasion resistance. Nano-layer thickness was found to be a critical performance factor, balancing comfort and protection. These results may be useful for developing low-cost, eco-friendly, and versatile protective products for medical application.

## 1. Introduction

Functional textiles with moisture-wicking (also called one-way water transport) performance that can provide comfortable functionality in hot and humid environments have received increasing attention [1,2]. The synergistic effects of both surface wettability and structural features, such as single-layered surface modification and double-layered hydrophobic/hydrophilic design, had been explored by scholars [3,4,5]. The former shows outstanding wicking performance because of its inherent hydrophilic nature. However, the latter exhibits maximum push-pull driving force to facilitate water-drop transport from one side to the other.

Recently, an electrospinning technique was reported to produce progressive wettability (wettability gradient) of Janus wettability directional water transport textiles based on adjusting the layer thickness. For example, Dong et al. [6] and Ding et al. [7] had reported directional water transport nano-fibrous membranes with various thicknesses by using an electrospinning method. Miao et al. [8] presented a facile design to prepare tri-layered nano-fibrous membranes with progressive wettability. Wu et al. [9] prepared a double-layer polyvinyl alcohol (PVA)/polyurethane (PU) nano-fibrous membrane by tailoring the thickness of a hydrophobic layer. On the basis of these studies, micro-/nano-structure design was used to promote the driving force. Wang et al. [10] developed polylactic acid (PLA)/cellulose acetate (CA) micro-/nano-fibrous membranes and found that the Murray structure (pores with different sizes) was favorable for improving the one-way water transport property. Xu et al. [11] designed a heterogeneous polypropylene (PP)/polyacrylonitrile (PAN) micro-/nano-fibrous membrane with excellent directional water transport properties. Unfortunately, although the above membranes showed outstanding directional water transport performance, such membranes were unable to offer additional protection. In our earlier work [12,13], we explored the effect of thickness gradient on breathable, moisture-permeable, and directional water transport performance; however, the mechanical and abrasion-resistance properties were insufficient. Generally, balancing the comfortability and protective performance of the versatile functional membranes was a huge challenge.

In the current work, directional water transport membranes were fabricated with excellent breathable, moisture-permeable, and abrasion-resistant performance. PLA and TPU were used as the outer and inner layers in the membrane to promote the push-pull effect. A needle punch/hot press electrospinning method was employed to form the fibrous composite membranes. This study differs from our previous work [12,13] on micro-/nano-structure. Nano-layer thickness was used to tailor the channel length in the micro-/nano-membrane matrix. It was found that thickness was a key factor balancing the breathable, moisture-permeable, abrasion resistant, and directional water transport performance. The prepared membranes indicate a promising utility for functional and medical moisture-wicking textiles.

## 2. Materials and Methods

### 2.1. Materials

Thermoplastic polyurethanes (TPU, EG-93A, Lubrizol Corp. Shanghai, China). LiCl and N, N-dimethylformamide (DMF) were obtained from Tianjin Kermel Chemical Reagent Co., Ltd (Tianjin, China). PLA fibers and low-melting PLA fibers (donated as LMPLA) were procured from Huvis Chemical Fiber Corp., Seoul, Korea.

### 2.2. Preparation of Bi-Layered PLA/TPU Membranes

**Figure 1:**
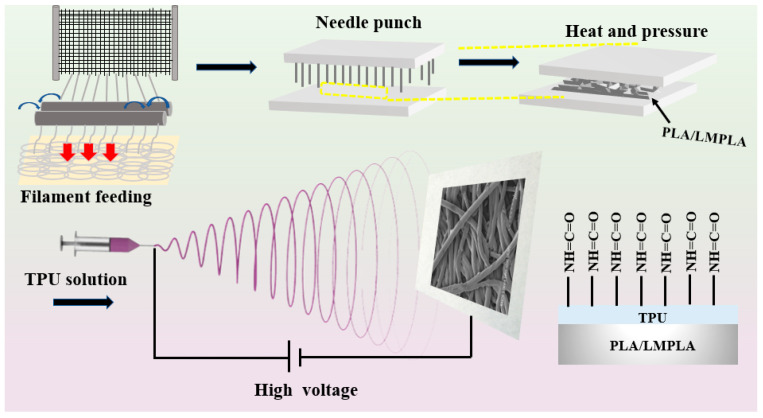
Preparation process of micro-/nano-composite membranes.

To begin with, PLA and LMPLA fibers were blended at 60/40, then the mixtures underwent opening, blending, laminating and needle-punching, successively, producing PLA/LMPLA micro-fibrous membranes with an areal weight and thickness of 150 ± 10 g/m^2^, and 300 ± 10 μm. The needle punch had a depth of 12 mm, needle diameter of 1 mm and specified stroke frequencies of 200 needles/min. Then, thermal bonding points were formed through hot-press treatment. Simultaneously, 0.0007 g LiCl, were dissolved in 100 g DMF solution under ultrasonic dispersion for 60 min to form a homogenous dispersion. Next, 1.8 g TPU was added to the above solution at 80 °C for 4 h. Third, the micro-/nano- fibrous membrane was obtained via electrospinning under a voltage of 45 kV, flow rate of 0.8 mL/h, and distance of 15 cm. The resulting composite membranes were named PLA-TPU-x, where x represents TPU thickness of 0, 8, 16, 32, and 64 μm, as depicted in Figure 1.

### 2.3. Measurements and Characterizations

A scanning electron microscope energy spectrum (SEM, S-4800, HITACHI, Tokyo, Japan) was taken to observe the morphology. Chemical structure was confirmed through FTIR spectrometer (NICOLET iS10, Thermo Fisher Scientific, Waltham, MA, USA). Wettability was evaluated using a water contact angle (WCA) instrument (JC2000DM, Shanghai Zhongchen Digital Testers, Shanghai, China). The air permeability of the samples was measured using an automatic air-permeability instrument (TEXTEST FX3300, Schwerzenbach, Switzerland). Each sample was 20 cm × 20 cm in size. Porosity of membranes was calculated based on the following Equation [14]:

(1) $$\mathrm{Porosity}=\left(1-\frac{m}{t\times S\times \rho }\right)\times 100\%$$

where *m*, *t*, *ρ*, and *S* represent the mass, thickness, density, and area per unit of measured membrane, respectively.

Water vapor transmission rate (WVTR) was measured according to GB/T12704.1 standards with a temperature of 38 °C, and relative humidity of 90%. The fabric samples were cut into circles 70 mm in diameter. The one-way water transport tests were carried on a moisture management tester (MMT, SDL ATLAS). The salted water was consistently dropped for 20 s on the membranes in accordance with AATCC195. The diameter of the sample was 8 cm. One-way transport index (also called *R* value) was calculated according to the Equation [15]:

(2) $$R=\frac{1}{T_{0}}\int \left[U_{b}(T)-U_{t}(T)\right]\;dT$$

where *U_b_*(*T*) and *U*_t_(*T*) represent waterhac content of bottom and top layer in the MMT, respectively. *T*_0_ represents the water feeding time.

**Figure 2:**
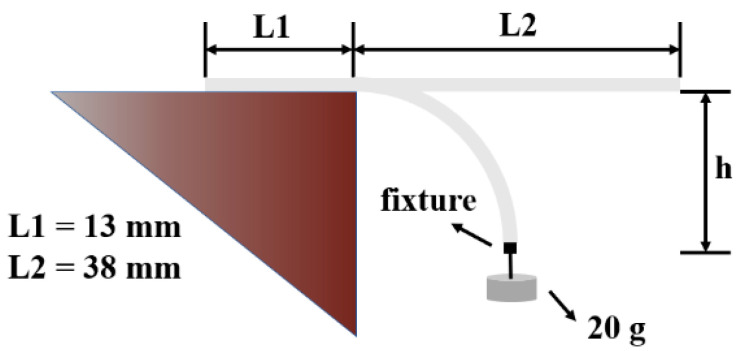
Flexibility test diagram.

Tensile strength was evaluated using an Instron 5565 (Instron, Boston, MA, USA), as specified in ASTM D5035-11. A sample with a size of 18 cm × 2.54 cm was mounted in the test instrument. Abrasion resistance was performed to evaluate durability in accordance with ASTM D3884-2013 standard. Each sample had a specified diameter of 80 mm. Flexibility of the membranes was evaluated by the cantilever beam method, as described by Zhang et al. [16] (Figure 2). The sample was cut into 50 mm × 51 mm, where L1:L2 = 13:38, and one end was fixed, while a weight of 20 g was hung from the other end. The sagging height (h) of the sample was measured to evaluate the flexibility of the membranes.

## 3. Results and Discussions

### 3.1. Morphology and Diameter Distribution of the Fibrous Membranes

**Figure 3:**
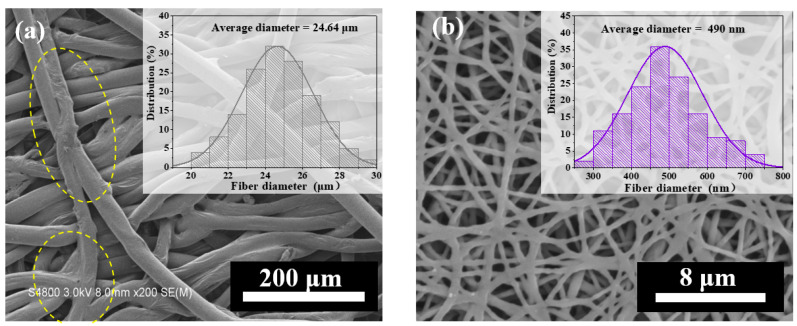
SEM image and diameter distribution of micro-/nano-composite membranes: (**a**) PLA side, (**b**) TPU side.

Figure 3 shows SEM images of the composite membrane. The membrane obtained by introducing the TPU nano-layer onto the PLA/LMPLA micro-layer surface. In Figure 3a, the PLA and LMPLA fibers were entangled via needle punching and exhibited similar morphology. Pure PLA fibers after hot press were randomly aligned without thermal bonding points because pure PLA fibers have a higher melting temperature than the hot press temperature. The addition of LMPLA presented the thermal bonding points after the hot press. This result was attributed to the thermal bonding temperature exceeding the melting temperature of the LMPLA sheath. The sheath was softened and melted, accordingly [17]. Thus, hot bonding points were formed to firmly bond the adjacent fibers. As presented in Figure 3b, the TPU side of the composite membrane exhibited randomly aligned and uneven staple fibers which could act as an interconnected passageway for vapor transmission. It is clearly observed from Figure 3 that the average diameters of the TPU and PLA/LMPLA fibers were 490 nm and 24.64 μm, respectively. The results confirmed the formation of micro-/nano-fibrous channel structure.

### 3.2. Surface Chemistry Structure of the Fibrous Membranes

**Figure 4:**
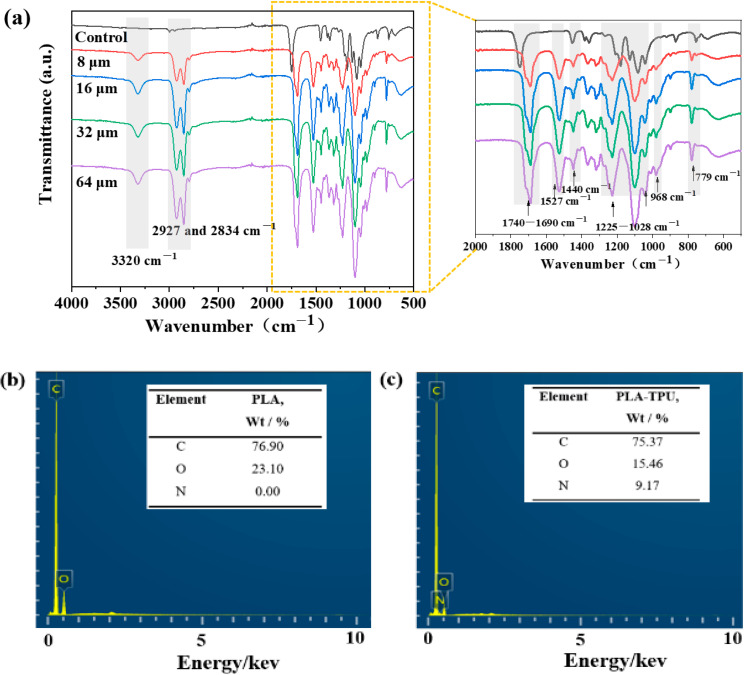
(**a**) FTIR spectrum of the control and composite membranes with various TPU thickness; EDS spectrum of (**b**) the control and (**c**) PLA-TPU-16 composite membranes.

Figure 4a shows the FTIR spectrum of the composite membranes with different TPU thickness. The characteristic peaks occurred at 2927 cm^−1^ and 2834 cm^−1^ for the stretching vibration of the alkane group [18]. The peaks related to the –C=O group were observed at 1690–1740 cm^−1^ [19]. The peaks at 1028–1225 cm^−1^ originated from –C–O stretching vibration [20]. –C–C stretching occurred at 968 cm^−1^ [8]. The bands at 779 cm^−1^ corresponded to the –C–H vibrations [21]. New characteristic peaks occurred at 3320 cm^−1^ and 1527 cm^−1^ for the stretching vibration of –N–H and –C–N–H [22]. The characteristic peaks on the spectra became gradually stronger, indicating an increased thickness of TPU. Figure 4b,c showed the element distribution of the fibrous membrane as two C and O elements belonging to micro-fibrous membranes, which were 76.9 and 23.1 wt%, respectively. Moreover, C, O, and N were homogeneously distributed along the PLA-TPU micro-/nano-fibrous composite membranes at 75.37, 15.46, and 9.17 wt%, indicating that the TPU nano layer was successfully deposited onto the PLA/LMPLA micro layer.

We have given careful consideration to the criticisms raised.

### 3.3. Wetting Behavior of the Fibrous Membranes

**Figure 5:**
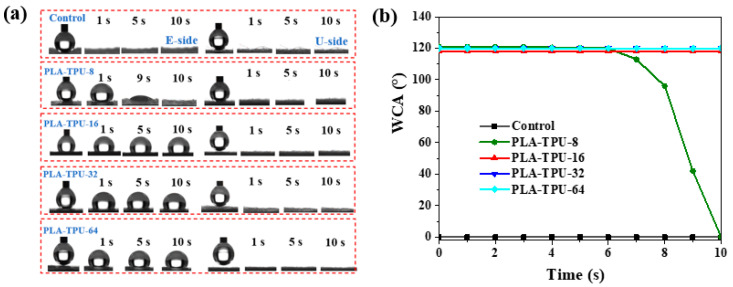
WCA of micro-/nano-composite membranes as related to different TPU layer thickness, (**a**) The dynamic photographs, and (**b**) WCA, water dropped onto TPU side.

The wettability of the micro-/nano-fibrous composite membranes with different TPU thicknesses was evaluated by WCA. Chemical composition and microstructure were key factors in determining wettability [8,23]. The nanolayer on the electrospinning side (referred as “E-side”) presented hydrophobicity, whereas the un-electrosprayed side (referred as “U-side”) maintained its hydrophilic property. Figure 5 shows that the hydrophobic performance of the TPU side was associated with the layer thickness. When the water was dropped onto the control membranes, the different sides of the membranes showed excellent hydrophilicity due to their hydrophilic property. For the PLA-TPU-8 composite membranes, the U side exhibited a similar hydrophilic property. Significantly, the WCA of the nano-fibrous side (E side) were 121°, 120.5°, 96°, 42°, and 0° at 1 s, 5 s, 7 s, 9 s, and 10 s. Furthermore, when the nano-fibrous layer thickness was in the range between 16 and 64 μm, the WCA of the E side was 120° within 10 s because of the increasing of the accumulating density. Notably, it is clearly found that water droplets on the U side were quickly spread out within 1 s. The results indicate that the composite membranes provide asymmetrical wetting performance and the TPU nano-layer thickness played a beneficial role in building the Janus structure.

### 3.4. Tensile Strength, Flexibility and Abrasion Resistance of the Fibrous Membranes

**Figure 6:**
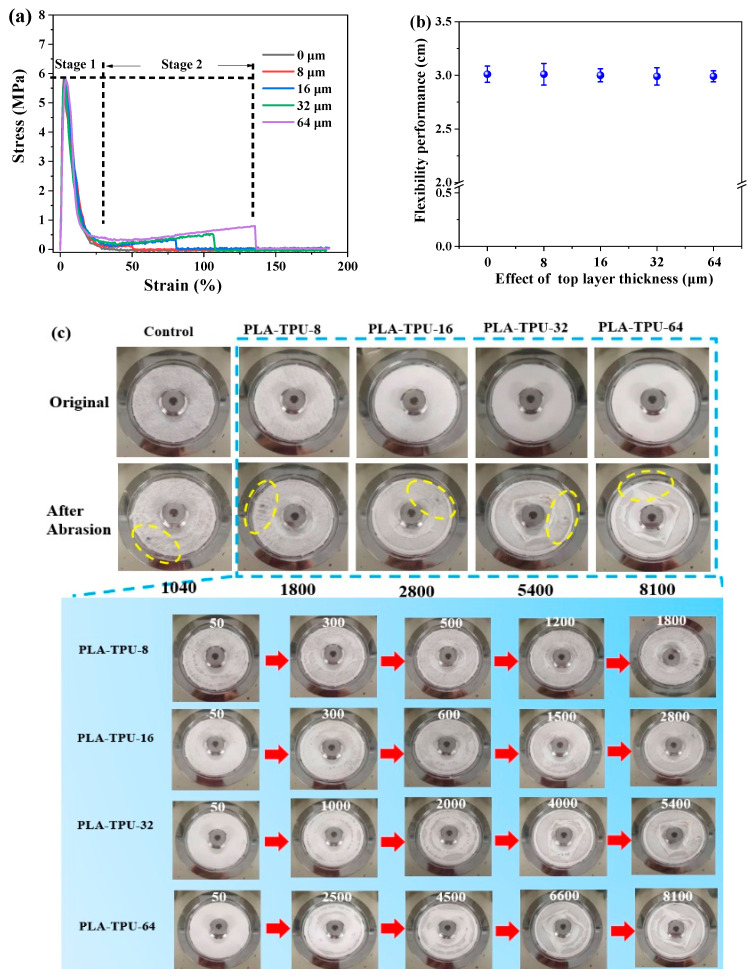
(**a**) stress–strain curves, (**b**) flexibility, and (**c**) abrasion resistance of micro-/nano-fibrous membranes as related to different TPU thicknesses.

Figure 6 shows the mechanical performance of micro-/nano-fibrous composite membranes, as related to TPU layer thickness. Figure 6a shows that composite membranes exhibit a double fracture mechanism. More interestingly, the thickness of the TPU layer showed negligible influence on the first tensile strength fracture because of the similar mass ratio of PLA and LMPLA. Furthermore, a rise in the TPU layer thickness improved the tensile strength of membranes significantly because the accumulating density of the TPU fibers was increased, which decreased the defect of the TPU nano-fibrous membrane and enabled the membranes to withstand an additional load.

Figure 6b demonstrates the effect of the TPU layer thickness on the flexibility of micro-/nano-fibrous composite membranes. The flexibility of the membranes was proportional to the sag height (h). The control membranes showed flexibility of 3.01 cm, and no evident height change of the composite membranes was observed, owing to flexibility of the TPU membranes [23,24]. Figure 6c shows the abrasion resistance results. Abrasion resistance of the control micro-fibrous membrane was relatively good (1040 cycles) because of the introduction of LMPLA, which enhances the entanglement and compactness among the adjacent fibers. Moreover, there was an obvious increase from 1800 cycles to 8100 cycles of abrasion resistance with TPU layer thickness from 8 μm to 64 μm. Notably, yellow line represented the damage morphology. Morphology of samples under different abrasion cycles could be seen in pictures around the red arrows. Benefitting from the TPU layer, the failure transformation increased from once to twice. Compared with the control membranes, the abrasion resistance of composite membranes was significantly improved, which corresponded to the thickness transformation [14]. The results were consistent with the tensile strength evaluation.

### 3.5. Moisture Permeability and Air Permeability of the Fibrous Membranes

**Figure 7:**
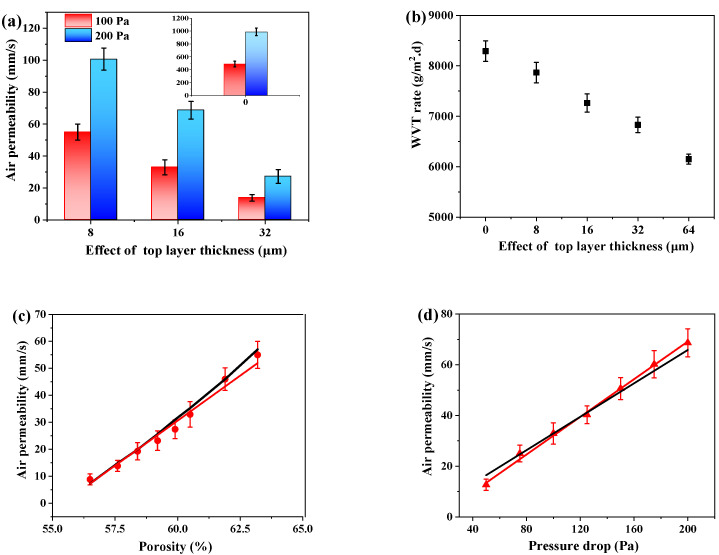

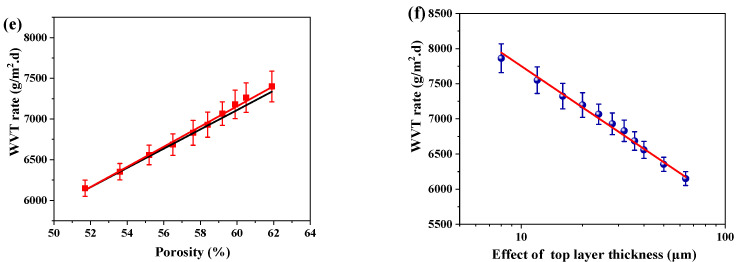
(**a**) air permeability and (**b**) WVT rate of a micro/-nano-composite membrane in relation to different TPU thicknesses; the linear relationship of PLA-PU-16 between (**c**) air permeability and porosity, (**d**) air permeability and pressure drop, (**e**) WVT rate and porosity, (**f**) WVT rate and thickness.

Considering outdoor textiles are intended to provide comfortable protection, moisture-permeable and breathable performance are indispensable for composite membranes [25,26,27]. The comfortable performance was evaluated by air permeability and WVT rate, as illustrated in Figure 7. According to previous literature [28,29,30], air transport belongs to the convection process, with air permeability expressed as Darcy’s law and shown in Equations (3)–(5):

(3) $$v=\frac{\Delta p\cdot k_{1}}{\mu \cdot L}.\rho .g$$

(4) $$k_{1}=\frac{\varepsilon^{3}}{k_{k}\cdot A_{0}^{2}\cdot {\left(1-\varepsilon \right)}^{2}}$$

(5) $$v=\frac{\Delta p\cdot \rho \cdot g\cdot \varepsilon^{3}}{\mu \cdot L\cdot k_{k}\cdot A_{0}^{2}\cdot {\left(1-\varepsilon \right)}^{2}}$$

where *v* is air permeability, *ρ*, *g*, and *μ*, are density, gravitational acceleration, and air viscosity, Δ*P* is the pressure drop, *k_k_* and *k*_1_ are the Kozeny constant and intrinsic permeability of membranes, respectively. *A*_0_, *L*, and *ε* are specific surface area, length, and porosity.

Compared with air permeability, the driving force of the WVT rate was the moisture concentration difference. Fick’s law could be used to explain the water vapor transmission [19,31]. WVT rate was determined on the basis of Equation (6):

(6) $$WVT\;rate=D.\frac{\varepsilon }{\tau }.\frac{\partial_{c}}{\partial_{x}}$$

In which *D* stands for the water diffusion coefficient. *τ*, *ε*, and $\partial_{c}/\partial_{x}$ stand for the tortuosity factor, porosity, and the water vapor concentration gradient, respectively.

Above these mechanisms, the relationship between comfortable performance and each parameter was investigated. Figure 7a shows the air permeability of micro-/nano-fibrous composite membranes in relation to various TPU layer thicknesses. The control membranes showed outstanding breathable performance (489 mm/s) because of the porous structure of the microfiber. However, the micro-/nano-fibrous membranes demonstrated decreased breathability, owing to the increment of the accumulating density and channel length [13,32]. Notably, the breathability results showed that air permeability with a pressure drop of 200 Pa was twice as high as that of the breathability results with a pressure drop of 100 Pa. This finding was supported by the results shown in Figure 7c,d. Figure 7c shows that the porosity had a linear relationship to the air permeability:

*v* (mm/s) = a × P (%) + b (7)

Therein, the black and red solid lines represent the theoretical calculation result according to Equation (5) and experimental results, respectively. The fitting R-square was 0.98. Namely, the theoretical calculation result was consistent with the experimental results. On the other hand, Figure 7d shows the linear relationship between air permeability (*v*) and pressure drop (P), which could be described according to Equation (8):

*v* (mm/s) = a × P (Pa) + b (8)

The fitting *R*-square was 0.99. Experimental results were in agreement with Darcy’s law. Similarly, the WVT rate decreased from 7865.83 g/m^2^·d to 6150.24 g/m^2^·d as the TPU layer thickness increased. Control micro-fibrous membranes provided greater water vapor transmission capability (8290.97 g/m^2^·d) than micro-/nano-fibrous composite membranes, which could be attributed to the decreased void among the adjacent microfibers, as discussed in Figure 3. With the introduction of LMPLA, an appropriate amount of thermal bonding points appeared between the fibers. The diameter of micron fiber was significantly larger than that of nanometer fiber (Figure 3). According to the previous studies, the macro-porous structure could be facilely regulated by controlling the fiber diameter and accumulating density [30,33,34,35]. In comparison with the micro-fibrous membranes, the nano-fibrous membranes had thinner fiber diameter and smaller pore size. The smaller the diameter, the higher the accumulating density, and vice versa. The micro-/nano-composite membranes were physically bonded structures, which could increase the accumulating density. Thus, increasing the TPU nano layer spinning time had an adverse effect on the water vapor transport properties. As a result, the porosity of the composite membranes decreased and the transport resistance elevated. Figure 7e shows that the WVT rate of the composite membranes decreased from 7400.24 g/m^2^·d to 6150.24 g/m^2^·d as the porosity decreased from 61 to 51%. The experimental results were in accordance with Fick’s law (Equation (6)). The WVT rate and porosity showed a positive linear correlation. The fitting R-square was 0.99; there was no difference between theoretical data and experiment results. It is worth noting that the WVT rate was decreased with the increment of TPU thickness on a logarithmic scale (Figure 7f).

### 3.6. Directional Water Transport of the Fibrous Membranes

**Figure 8:**
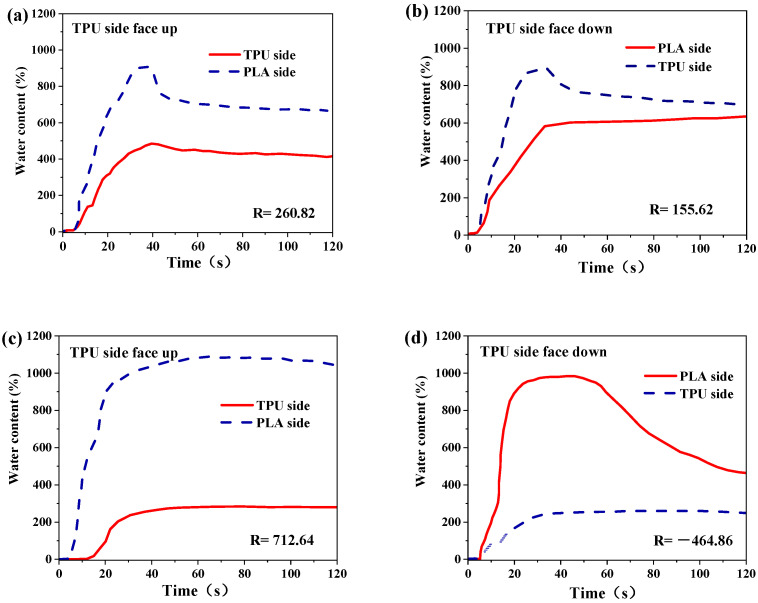

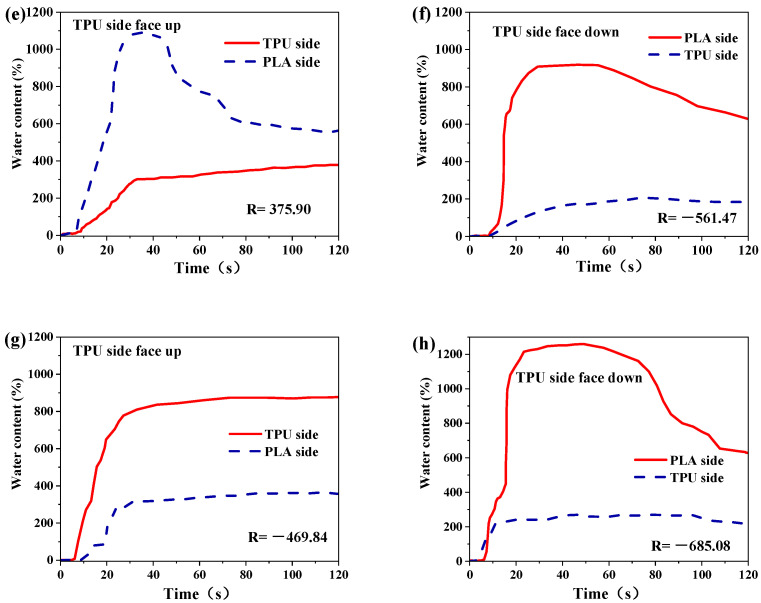
MMT results on composite membranes under different TPU thicknesses: (**a**,**b**) 8 μm, (**c**,**d**) 16 μm, (**e**,**f**) 32 μm, (**g**,**h**) 64 μm. The water was dropped onto the different sides.

In order to further study the water transport behavior, a moisture management tester (MMT) was used to measure the water content. During testing, saline water (containing 0.9% NaCl) was dropped onto the different sides of the composite membranes, and the water content was recorded in the first 120 s. The MMT results were also compared for the two sides of the PLA-TPU-8 membrane (Figure 8a,b, respectively). The water content showed a similar initial increasing and then decreasing trend regardless of the side on which the water was dropped. In this case, the water content on the TPU surface reached about 260.82%, while the water content on the PLA side was around 155.62%. Membranes indicated obvious two-way transport features.

For the PLA-TPU-16 composite membranes, when the water was dropped onto the TPU side, the water content on both sides increased in the first 20 s owing to the water supplement. It can be seen from Figure 8c that the water content of the bottom side (micro- fibrous membranes) was always higher than the top layer, indicating that the water could be effectively transported from the TPU side to the PLA side. However, when the water was dropped onto the PLA side (Figure 8d), the water content on the top side reached about 1050%, while the water content on the TPU side was around 260%, indicating that water would remain in the top side. The micro-/nano-fibrous composite membranes exhibited one-way transport. This was attributed to the competition against hydrostatic pressure, hydrophobic force, and capillary force offered by the hydrophobic and hydrophilic layer. Asymmetric wettability had a positive effect on promoting water downward penetration. Meanwhile, for the PLA-TPU-32 composite membranes (Figure 8e,f), the difference in water content was smaller than that of the PLA-TPU-16 composite membranes, which could be attributed to the increasing TPU layer thickness, which enhanced the hydrophobic force and hindered further penetration of water. Thus, the *R* value decreased from 712.64 to 375.90%. For the PLA-TPU-64 micro-/nano-fibrous composite membranes, no matter onto which side water was dropped, the water content of the top side was always larger than that of the bottom side, exhibiting a similar trend (Figure 8g,h). The *R* values of the two sides were −469.84% and −685.08%, respectively, which shows a negative one-way transport index to offer a non-transport feature. The above results indicate that the wettability gradient and thickness gradient across fibrous composite membranes were the two main sources to trigger directional water transport behavior in water–air systems [12]. These results were supported by the WCA analysis.

**Figure 9:**
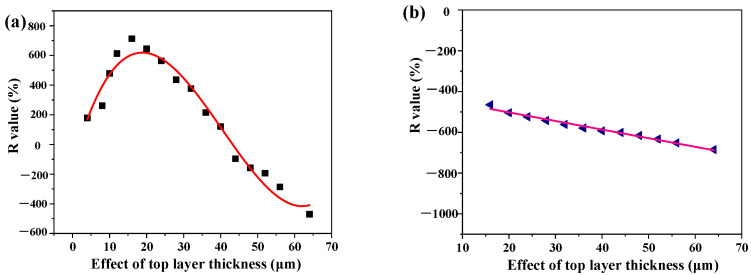
One-way transport index (*R* value) of composite membranes with different hydrophobic layer thickness: (**a**) water was dropped onto the hydrophobic side, (**b**) water was dropped onto the hydrophilic side.

Figure 9 shows the *R* value of the PLA-TPU-16 micro-/nano-fibrous composite membranes. Figure 9a shows that the *R* value was increased with increments of the TPU layer and then decreased to a negative value. When the TPU layer thickness ranged between 16 and 40 μm, the composite membranes exhibited one-way water transport behavior. There was a negative *R* value on the two sides following TPU layer thickness larger than 40 μm. The membranes showed no directional liquid transport abilities. When the thickness was smaller than 16 μm, the membranes displayed two-way water transport behavior. More interestingly, when the water was dropped onto the PLA side, we found a linear relation between the *R* value and TPU layer thickness.

**Figure 10:**
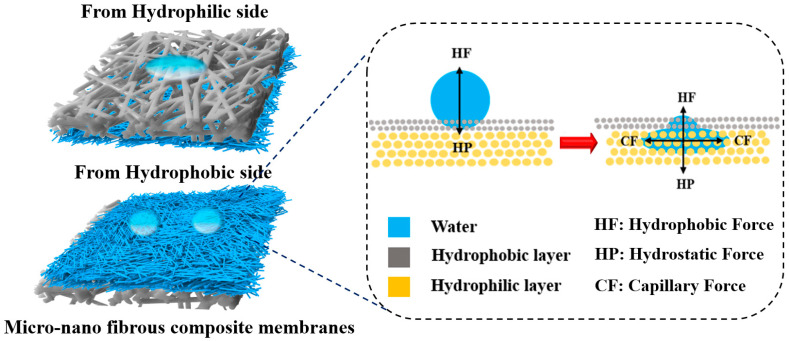
Schematic illustration of the water on the micro-/nano-fibrous composite membranes.

On the basis of these results, the possible directional water transport mechanism in micro-/nano-fibrous composite membranes was proposed, as seen in Figure 10. In this work, the driving force arising from the difference in the wettability with drive water dropped from the TPU side moved to the PLA side. From the TPU side, hydrostatic pressure and hydrophobic force pushed water cross toward the TPU layer. However, with TPU layer thickness larger than 40 μm, hydrophobic force makes the water droplet retain a Wenzel-Cassie state, which makes it less likely to penetrate the membranes. On the contrary, when water was dropped onto the PLA side, the capillary force triggered water to spread in all possible directions. The water droplet could not travel from the top side to the bottom side.

The functional and comfortable performance of the proposed directional transport micro-/nano-fibrous composite membranes were compared with the results of a previous study. As can be seen from Table 1, previous reported membranes exhibited unique unidirectional water transport, or moisture-permeable and breathable performance. By contrast, benefitting from the micro-/nano-porous structure, PLA-TPU micro-/nano-fibrous composite membranes showed greater moisture-permeable and breathable performance (7375.94 g/m^2^·d, 37.9 mm/s), excellent one-way water transport capability (712.64%), and a higher abrasion-resistant protective property (2800 cycles). The eco-friendly composite membrane in this work provides high-performance comfort, protection, and versatility, suggesting a promising candidate for all types of potential applications in comfortable directional water transport textiles.

## 4. Conclusions

The proposed PLA-TPU micro/-nano-fibrous composite membranes were successfully generated through needle punch, hot press, and electrospinning. The impacts of TPU layer thickness on the surface chemical structure, comfortability, and protective properties were evaluated. Consequently, when the TPU layer thickness ranged between 16 and 40 μm, the resulting composite membranes had a high *R* value of 712.64%, a moisture permeability of 7375.94 g/m^2^·d, a breathability of 37.9 mm/s, and an abrasion resistance of 2800 cycles. Furthermore, air permeability and WVT rate were in accordance with Darcy’s Law and Fick’s Law, respectively. It is notable that TPU layer thickness plays important roles in balancing the comfortability and protective properties. These novel understandings may be useful for designing high-performance moisture-wicking textiles for various applications.

## Figures and Tables

**Table 1:** Summary of the transport performances of this study and previous studies.

Specimen	Modification Method	WVT Rate, g/m^2^·d	Air Permeability, mm/s	R, %	Ref.
PLA-TPU	Electrospinning	7375.94	37.9	712.64	This work
TPU/TBAC-TPU	Electrospinning	2170	1.16	34.93	[36]
CA-PU	Electrospinning	—	—	297	[37]
ZnO-PVDF/PAN	Electrospinning	—	—	172.7	[38]
PAN/PDOPA-PS	Electrospinning	—	—	287.6244	[6]
CA-PAN/PVDF	Electrospinning	—	—	276.5283	[39]
Woven-PDA/PPy	Dip-coating	—	—	50.3514	[1]
PAN-HPAN-PU	Electrospinning	—	—	1021	[8]
PU/FPU-CNTs	Electrospinning	9200	<20	—	[30]
FPU/PAN/PVB	Electrospinning	9600	<20	—	[35]
PAN/ASO/SiO_2_	Electrospinning	11,400	<20	—	[40]
Commercial PU membrane	Tape casting	7200	<5	—	[23]
Gore-Tex membrane	Tape casting	5000	2.4	—	[41]

## Data Availability

Not applicable.
